# Structured reflection increases intentions to reduce other people’s health risks during COVID-19

**DOI:** 10.1093/pnasnexus/pgac218

**Published:** 2022-10-03

**Authors:** Jairo Ramos, Marrissa D Grant, Stephan Dickert, Kimin Eom, Alex Flores, Gabriela M Jiga-Boy, Tehila Kogut, Marcus Mayorga, Eric J Pedersen, Beatriz Pereira, Enrico Rubaltelli, David K Sherman, Paul Slovic, Daniel Västfjäll, Leaf Van Boven

**Affiliations:** Department of Psychology and Neuroscience, University of Colorado, Boulder, CO 80309, USA; Department of Psychology and Neuroscience, University of Colorado, Boulder, CO 80309, USA; School of Business and Management, Queen Mary University of London, London E1 4N, UK; Department of Psychology, University of Klagenfurt, 9020 Klagenfurt, Austria; School of Social Sciences, Singapore Management University, 81 Victoria St, Singapore 188065; Department of Psychology and Neuroscience, University of Colorado, Boulder, CO 80309, USA; School of Psychology, Swansea University, Singleton Park, Swansea SA2 8PP, UK; Department of Education, Ben-Gurion University of the Negev, 653, Beer-Sheva 8410501, Israel; Decision Research; University of Oregon, 1585 E, 13th Ave, Eugene, OR 97403, USA; Department of Psychology and Neuroscience, University of Colorado, Boulder, CO 80309, USA; Department of Marketing, Ivy College of Business, Iowa State University, Gerdin Business Bldg, 2167 Union Dr, Ames, IA 50011, USA; Department of Developmental and Social Psychology, University of Padua, Via 8 Febbraio, 2-35122 Padova, Italy; Department of Psychological and Brain Sciences, University of California, Santa Barbara, CA 93106-9660, USA; Decision Research; University of Oregon, 1585 E, 13th Ave, Eugene, OR 97403, USA; Department of Behavioural Sciences and Learning, Division of Psychology, Linköping University, SE-581 83 Linköping, Sweden; Department of Psychology and Neuroscience, University of Colorado, Boulder, CO 80309, USA

**Keywords:** COVID-19, decision making, boost, nudge, structured reflection

## Abstract

People believe they should consider how their behavior might negatively impact other people, Yet their behavior often increases others’ health risks. This creates challenges for managing public health crises like the COVID-19 pandemic. We examined a procedure wherein people reflect on their personal criteria regarding how their behavior impacts others’ health risks. We expected structured reflection to increase people's intentions and decisions to reduce others’ health risks. Structured reflection increases attention to others’ health risks and the correspondence between people's personal criteria and behavioral intentions. In four experiments during COVID-19, people (*N*  = 12,995) reported their personal criteria about how much specific attributes, including the impact on others’ health risks, should influence their behavior. Compared with control conditions, people who engaged in structured reflection reported greater intentions to reduce business capacity (experiment 1) and avoid large social gatherings (experiments 2 and 3). They also donated more to provide vaccines to refugees (experiment 4). These effects emerged across seven countries that varied in collectivism and COVID-19 case rates (experiments 1 and 2). Structured reflection was distinct from instructions to carefully deliberate (experiment 3). Structured reflection increased the correlation between personal criteria and behavioral intentions (experiments 1 and 3). And structured reflection increased donations more among people who scored lower in cognitive reflection compared with those who scored higher in cognitive reflection (experiment 4). These findings suggest that structured reflection can effectively increase behaviors to reduce public health risks.

Significance Statement Addressing societal challenges like the COVID-19 pandemic requires the public to voluntarily behave in ways that minimize other people's risks. The results of four experiments with participants from seven countries demonstrated that people are more inclined to reduce other people's COVID-19 risks after reflecting on their personal beliefs about how much such considerations should influence their behavior. These findings suggest that communication during public health crises can be effectively structured to encourage behaviors that minimize public health risks.

## Introduction

Addressing societal challenges, from climate change to pandemics, requires voluntary behaviors to minimize others’ risks. Although most people believe they should consider whether their behavior might negatively impact others, people often behave in ways that increase others’ health risks. During the COVID-19 pandemic, for example, public health guidance advised people to limit social contact. Although people generally endorsed such guidelines, adherence was often limited (1). Intentions influence behavior (2, 3), so it is important to examine procedures to increase people's intentions to minimize others’ health risks, consistent with their personal beliefs.

We suggest that a procedure of structured reflection on personal criteria increases people's intentions and decisions to reduce others’ health risks. This is because people are typically inattentive to public health risks and the consistency between their personal criteria and their behaviors. Structured reflection reduces inattention.

### Underweighting public health risks

People's intentions and behaviors, like other preferences, are constructed, in part, by the context in which they report them (4–6). Contextual factors can focus attention on prominent attributes, leading people to underweight nonfocal attributes—even when, upon reflection, they believe that those attributes are at least as important (7–12). Because personal consequences are typically focal (13–17), people weigh them more than the nonfocal consequences of their behavior for other people.

Consider the decision to attend a large gathering during the COVID-19 pandemic. Even though people might think it is important to minimize the risk of spreading disease, their desire to socialize with others may be more prominent, increasing intentions to socialize despite the health risks imposed on other people. People may therefore underweight public health risks relative to their personal criteria when forming intentions and making decisions.

### Structured reflection on personal criteria

We propose that a procedure of structured reflection on personal criteria increases people's intentions to reduce others’ health risks. Oxford Languages defines a criterion as “a principle or standard by which something may be judged or decided.” Personal criteria are personal principles and standards about how much specific attributes should influence behavior (18–22). Reflection entails serious thought and consideration of those criteria, similar to the self-reflection on cognitive processes (23, 24), past experiences (25), and personal values (26) that can influence judgments and decisions. The proposed procedure provides structure by asking people to reflect on their criteria about predefined attributes, such as how their behavior impacts others’ health risks. Providing structure is an important component of decision analysis because it requires people to evaluate multiple attributes (27, 28). Structured reflection thus leads people to construe their intentions and behaviors relative to their personal criteria (29, 30).

Structured reflection on personal criteria operates through at least two processes. First, it increases attention to public health risks, which directly increases their weight (31–36). Second, structured reflection increases awareness of the consistency between personal criteria and behavior (28). People are motivated to maintain consistency (31–36), so such awareness prompts people to change behavior to align with their personal criteria. And because most people believe they should not impose risks on others (37–39), structured reflection should increase intentions to reduce others’ risks.

### Cross-country comparisons

Experiments 1 and 2 included national samples from seven countries (Supplementary Material Table S1). We selected these countries to increase generalizability (40–42) by examining a range of cultures and pandemic policies. Structured reflection might have a larger effect in more individualistic cultures, for example, where people are more motivated to maintain consistency (43–47). And structured reflection might have a larger effect in countries with less stringent COVID-19 policies, where voluntary behavioral intentions are most relevant.

### Overview of experiments

Participants considered scenarios reflecting tradeoffs between two attributes. One nonfocal attribute involved considerations of other people's health risks. The other, more focal attribute differed across experiments. Experiments 1 and 4 involved tradeoffs between minimizing others’ risks and economic benefits. Experiment 2 involved personal risks (as opposed to others’ risks) of COVID-19. Experiment 3 involved friends’ and family's enjoyment of a social gathering. The correlations between personal criteria varied across experiments 1 to 4 (0.07, 0.74, −0.12, and 0.36, respectively), allowing comparison of structured reflection on both correlated and uncorrelated criteria.

We randomly assigned people to reflect on their personal criteria, either before or after reporting their intentions or decisions. We hypothesized that structured reflection would increase intentions and decisions that reduce other people's COVID-19 risk.

## Results: experiment 1: reducing restaurant capacity

Participants (*N*  = 6,188) imagined that they owned a small restaurant business and were considering reducing capacity by 50% to minimize the spread of COVID-19. However, reducing capacity would also result in significant income loss. We conducted the experiment in August 2020, following the first large wave of the pandemic, when many countries were easing restrictions on economic activity and before vaccinations were available. In the control condition, participants stated their intention to reduce capacity directly after reading the scenario (1 = * Extremely unlikely*; 7 = *Extremely likely*). In the structured reflection condition, participants first reported how much (1 = *Not at all*; 7 = *Very much*) they believed that two attributes should influence their decision: the risk that COVID-19 would spread to other people and the economic losses from reducing capacity. Participants in the control condition reported their criteria after reporting their intentions.

Participants reported that the risk of spreading COVID-19 (*M = *5.09, *SD* = 1.55) should influence their decisions more than economic losses (*M = *4.17, *SD *= 1.64; *B* = −0.91, *SE* = 0.19, *CI* = [−1.28, −0.53], *P*  = 0.003). The two criterion ratings were slightly correlated ( *r* = 0.07, *P* < 0.001) and the difference did not differ significantly between conditions (*B* = −0.03, *SE* = 0.05, *CI* = [−0.13, 0.08], *P *= 0.621).

As predicted, when we categorized intentions relative to the scale midpoint, participants in the structured reflection condition were more likely to report intentions to reduce capacity (72%) compared with the control condition (65%; *B* = 0.33, *SE* = 0.10, *OR* = 1.39, *CI* = [1.14, 1.69], *P*  = 0.001, Supplementary Material Table S3). Using a mixed effect model with random intercepts for country, we regressed continuous ratings of intentions to reduce capacity on condition (control = -½ and structured reflection = ½), ratings of each personal criteria (grand mean-centered), and interactions between condition and each criterion. Participants in the structured reflection condition intended to reduce business capacity ( *M* = 5.19, *SD* = 1.64) more than did participants in the control condition (*M* = 4.91, *SD *= 1.73; *B* = 0.26, *SE* = 0.07, *CI* = [0.11, 0.40], *P* = 0.014, Supplemenatry Material Table S4).

There was also a significant interaction between condition and personal criteria of reducing the risk of spreading COVID-19 (*B *= 0.08, *SE *= 0.02, *CI* = [0.03, 0.13], *P *= 0.001), which more strongly predicted intention to reduce capacity in the structured reflection condition (*B* = 0.55, *SE *= 0.02, *CI* = [0.51, 0.58], *P* < 0.001) than in the control condition (*B *= 0.47, *SE *= 0.02, *CI* = [0.43, 0.50], *P *< 0.001). There was an analogous interaction between condition and personal criteria of economic considerations (*B* = −0.05, *SE* = 0.02, *CI* = [−0.10, −0.01], *P *= 0.018), which more strongly predicted intention to reduce capacity in the structured reflection condition (*B* = −0.17, *SE* = 0.02, *CI* = [−0.20, −0.14], *P *< 0.001) than in the control condition (*B* = −0.11, *SE *= 0.02, *CI* = [−0.15, −0.08], *P *< 0.001). Structured reflection thus increased intentions to reduce business capacity and increased the correlation between intentions and personal criteria.

## Results: experiment 2: attending a social gathering

People weigh prominent personal risks more than less prominent risks their behavior imposes on other people (13–17, 48–50). The societal costs of such weighting can be devastating even if personal risks are low, as when a modest percentage increase in COVID-19 hospitalizations overwhelms medical systems. Furthermore, COVID-19’s aerosol spread meant that social contact posed risks to many more people than oneself (51–53). Structured reflection on personal criteria might therefore reduce intentions to attend a large social gathering when considering the COVID-19 risks imposed on other people.

In 2020 August, we asked participants (from the same cross-country sample, who did not complete experiment 1, *N *= 6,035) whether they would cancel their plans to attend a gathering with 50 friends following months of social isolation. They read that the local government cautioned against meeting in groups greater than 10 to minimize the spread of COVID-19. Participants in the control condition reported the likelihood they would attend the gathering (1 = *Extremely*  *unlikely*; 7 = *Extremely likely*). Before reporting their likelihood, participants in the structured reflection condition reported their personal criteria about how much (1 = *Not at all;* 7 = *Very much*) two attributes should influence their decision: the possibility of spreading COVID-19 to others and the possibility of personally experiencing severe symptoms. Control condition participants reported their criteria after reporting intentions.

Participants reported that both the risk of spreading COVID-19 (*M* = 5.36 and *SD* = 1.77) and the threat of experiencing severe symptoms (*M = *5.26 ,*SD *= 1.80) should influence their decision. The two ratings were strongly positively correlated (*r* = 0.74, *P *< 0.001). Neither rating differed across condition (spreading COVID-19, *B* = 0.07, *SE = 0*.07*, CI* = [−0.06, 0.20], *P *= 0.349; personal symptoms, *B* = 0.12, *SE = 0*.06*, CI* = [−0.01, 0.24], *P *= 0.123).

As predicted, when intentions were categorized relative to the scale midpoint, participants in the structured reflection condition were more likely to say they would not attend the gathering (72%) compared with the control condition (67%; *B* = 0.22, *SE* = 0.06, *OR* = 1.24, *CI* = [1.11, 1.39], *P *< 0.001, Supplementary Material Table S6). Using a mixed effects model with random intercepts for country, we regressed continuous intentions on condition (control = -½ and structured reflection = ½), ratings of each personal criteria (grand mean-centered), and the interactions. As predicted, participants in the structured reflection condition had lower intentions to attend the gathering (*M* = 2.55, *SD *= 1.81) compared with the control condition (*M* = 2.82, *SD *= 1.95; *B* = −0.20, *SE *= 0.04, *CI* = [−0.29, −0.12], *P *< 0.008, Supplemenatry Material Table S7). Structured reflection thus increased intentions to cancel a social gathering to minimize the risk of spreading COVID-19.

Participants’ intentions to attend the gathering were predicted both by their criteria regarding spreading COVID-19 (*B* = −0.32, *SE* = 0.02, *CI* = [−0.36 , −0.28], *P* < 0.001) and regarding personal symptoms (*B* = −0.17, *SE* = 0.02, *CI* = [−0.20, −0.13], *P* < 0.001). Unlike experiments 1 and 3, however, these correlations did not differ significantly across condition (risk of spreading interaction, *B* = −0.06, *SE =* 0.04*, CI* = [−0.13, 0.01], *P *= 0.083; symptom severity interaction, *B* = 0.02, *SE =* 0.04*, CI* = [−0.05, 0.09], *P *= 0.521). The absence of a significant interaction might be because the criteria were strongly correlated, and people have difficulty assigning different decision weights to correlated cues (54).

## Results: cross-country comparisons in experiment 1 and experiment 2

Because we conducted our experiments in seven countries, we explored cross-country differences in the relationships between structured reflection, personal criteria, and behavioral intentions. First, we re-estimated the primary mixed effects models for both experiments, including as fixed effect predictors country-level collectivism (as measured by Hofstede's Cultural Dimensions model, with higher numbers indicating greater individualism, see the Supplementary Material Table S2; *M_Experiment__1_* = 63.41, *SD_Experiment__1_* = 23.30; *M_Experiment__2_* = 63.65, and *SD_Experiment__2_* = 22.72) along with interactions between individualism and structured reflection effects. Consistent with the notion that individualism is associated with greater motivation for consistency, the relationship between behavioral intentions and personal criteria to minimize others’ COVID-19 risk was larger in countries with relatively higher levels of individualism (grand mean-centered, experiment 1 interaction: *B *= 0.003, *SE* = 0.001, *CI* = [0.002, 0.004], *P* < 0.001; experiment 2 interaction: *B* = −0.005, *SE* = 0.001, *CI* = [−0.007, −0.004], *P* < 0.001, Supplementary Material Tables S5 and S8). Country-level individualism did not, however, moderate the effect of structured reflection on intentions (experiment 1 interaction, *B *= 0.000, *SE* = 0.003, *CI* = [−0.007 , 0.007], *P* = 0.986; experiment 2 interaction, *B* = −0.000, *SE* = 0.002, *CI* = [−0.005, 0.005], *P* = 0.959).

**Fig. 1.**
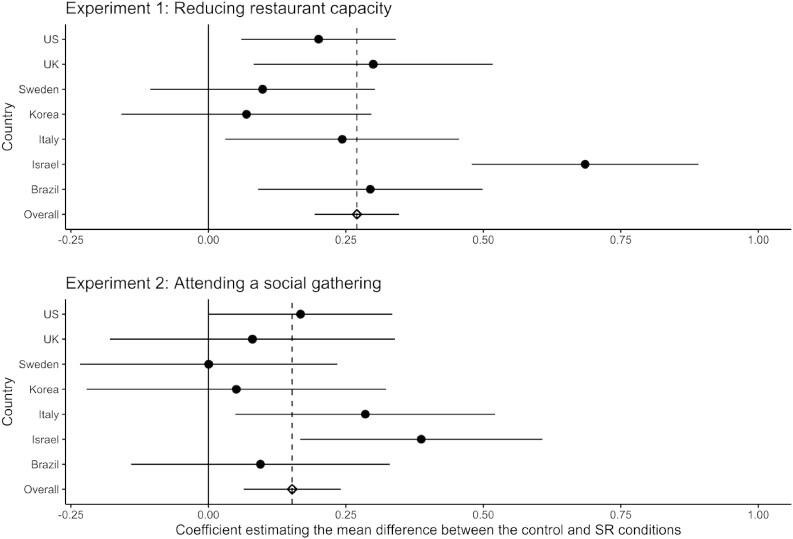
Estimated mean differences for each country for experiments 1 (reducing restaurant capacity; top panel) and 2 (intentions to attend a social gathering; bottom panel). The *x*-axis represents the estimated mean difference between the structured reflection and control conditions. These results come from regression models including condition, ratings of personal criteria, the interactions between condition and personal criteria, and the interactions between this set of predictors and six codes representing country differences. For ease of interpretation, the effects in experiment 2 are coded such that higher personal criteria indicate a greater reduction in intentions to attend a gathering. Vertical dashed lines represent point estimates for the fixed effect of structured personal criteria analysis across countries. Horizontal lines represent 95% *CI*s around the estimated effect size.

Second, we explored whether the effects differed significantly across countries. We reanalyzed the data from experiments 1 and 2, with fixed effects for country (Fig. 1). There was minimal cross-country variation, except for Israel, which emerged as the only consistently different country, exhibiting larger effects of structured reflection (experiment 1 *B* = 0.41, *SE* = 0.10, *CI* = [0.22 , 0.60], *P *< 0.001; experiment 2 *B* = −0.23, *SE* = 0.11, *CI* = [−0.44, −0.03], *P *= 0.026). At the time of the experiments, Israel had fewer restrictions and lower reported COVID-19 death and hospitalization rates than other countries (Supplementary Material Table S1). Israeli participants may therefore have been more tolerant of risky behaviors, reflected by the intentions of Israeli participants in the control conditions (Supplementary Material Table S2), and more open to interventions that reduce risky behaviors. These findings provide evidence for the cross-country generalizability of the finding that structured reflection increases intentions to minimize others’ health risks.

## Results: experiment 3: canceling a family Thanksgiving holiday

We reason that structured reflection operates by increasing attention to others’ health risks and the consistency between their behavior and personal criteria. Alternatively, structured reflection might prompt analytical deliberation (12, 55–60), which can lead people to assign greater weight to otherwise underweighted attributes. To address this alternative, we experimentally manipulated instructions to engage in deliberation.

Shortly before the November 2020 Thanksgiving holiday in the United States, participants (*N* = 2,420) considered whether to cancel a planned family gathering with more than 30 family members. The scenario described two attributes, the risk of spreading COVID-19 to others and the desire to socialize with family. Participants randomized to the control condition reported their intention to cancel the holiday (1 = *Extremely unlikely;* 7 = *Extremely likely*). Participants randomized to the deliberation condition were first asked to “consider your decision carefully and try to generate clear reasons for why you should or should not cancel the Thanksgiving gathering.” Participants in the structured reflection condition were first asked to report how much (1 = *Not at all;* 7 = *Extremely*) their decision should be influenced by “the likelihood that COVID-19 may spread among family members” and “the satisfaction of spending time with family members.” Participants in the control and deliberation conditions reported personal criteria after reporting their intentions to cancel the gathering.

Participants reported that the risk of spreading COVID-19 (*M* = 5.44, *SD *= 1.90) should influence their decision to cancel Thanksgiving more than the satisfaction of socializing (*M* = 4.03, *SD *= 1.90; *B* = 1.40, *SE* = 0.06, *CI* = [1.29, 1.52], *P *< 0.001). This difference was significant in all conditions (all *P*s < 0.001), although it was larger in the structured reflection condition (*M_diff_ *= 1.61, *SD_diff_ *= 2.74) than in the other conditions (deliberation, *M_diff_ *= 1.31, *SD_diff_ *= 2.85; control, *M_diff_ *= 1.30, *SD_diff_ *= 2.92; *B* = 0.31, *SE* = 0.12, *CI* = [0.07 , 0.55], *P *= 0.012, Supplementary Material Table S9). Because the regression analysis reported below includes criteria ratings and their interactions, it controls for these condition differences. Ratings of the two criteria were somewhat negatively correlated (*r* = −0.12 , *P* < 0.001).

We categorized intentions relative to the midpoint. As predicted, participants in the structured reflection condition were more likely to cancel the Thanksgiving holiday (65%) compared with the deliberation and control conditions (each 60%; *B* = −0.21, *SE* = 0.09, *OR* = 1.23, *CI* = [1.03, 1.47], *P* = 0.020, Supplementary Material Table S10). We regressed continuous intentions on condition contrast codes and their interactions with each criteria rating (grand mean-centered). Participants in the structured reflection condition reported they were more likely to cancel the Thanksgiving gathering (*M* = 5.22, *SD *= 2.04) compared with the average of deliberation (*M* = 4.93, *SD *= 2.16) and control conditions (*M* = 4.88, *SD *= 2.19; *B *= 0.15, *SE *= 0.06, *CI* = [0.03, 0.26], *P *= 0.015), which were not significantly different from each other (*B *< 0.01, *SE *= 0.07, *CI* = [−0.13, 0.14], *P = 0*.965, Supplementary Material Table S11).

Structured reflection increased the consistency between personal criteria and intentions to cancel the holiday. There was an interaction between the structured reflection condition contrast and criteria of spreading COVID-19 (*B* = 0.11, *SE *= 0.03, *CI* = [0.05, 0.18], *P *< 0.001). The criteria of spreading COVID-19 predicted intentions to cancel Thanksgiving more strongly in the structured reflection condition (*B* = 0.91, *SE *= 0.03, *CI* = [0.86, 0.97], *P *< 0.001) than in the average of deliberation (*B* = 0.77, *SE *= 0.02, *CI* = [0.72, 0.82], *P *< 0.001) and control conditions (*B* = 0.82, *SE *= 0.03, *CI* = [0.77 , 0.88], *P *< 0.001), which did not differ from each other (*B* = −0.05, *SE *= 0.04, *CI* = [−0.12, 0.02], *P *= 0.142). Criteria ratings of socializing negatively predicted intentions to cancel the holiday (*B* = −0.10, *SE *= 0.01, *CI* = [−0.13, −0.07], *P *< 0.001) but did not interact with condition (structured reflection compared with deliberation and control: *B* = −0.02, *SE *= 0.03, *CI* = [−0.08, 0.05], *P* = 0.610; deliberation compared with control: *B* = −0.03, *SE *= 0.04, *CI* = [−0.10, 0.04], *P* = 0.442). Structured reflection thus increased intentions to cancel Thanksgiving, increasing consistency with personal criteria that the risk of spreading COVID-19 should influence their decisions.

## Results: experiment 4: donating for humanitarian vaccines

We tested whether structured reflection would influence nonhypothetical decisions involving tradeoffs between personal benefit and reducing others’ health risks. Experiments 1 to 3 included purely hypothetical scenarios, which might have heightened the influence of experimental demand and social desirability considerations. Such concerns are diminished in decisions with financial consequences.

To deepen understanding of underlying processes, we also examined whether individual differences in cognitive reflection (23, 24) would moderate the effect of structured reflection. We hypothesize that structured reflection operates partly by prompting people to reflect on their decisions and the consistency with their personal criteria. This analysis implies that the manipulation of structured reflection would have a smaller effect among people with higher cognitive reflection scores, who are already predisposed to reflect.

Participants (*N*  = 1,080) in March and April, 2022, learned that they would be entered into a lottery for a ${\$}$100 cash prize in exchange for completing a survey. We asked participants to decide how much of their prize to donate to the International Rescue Committee’s (IRC’s) COVID-19 vaccination campaign, keeping the remainder as “personal economic relief funds.” The IRC is a nonprofit organization that provides COVID-19 vaccines to refugees with limited access to medical care. Participants learned that experimenters would allocate the ${\$}$100 as specified if they won the lottery. We reminded participants they were not required to donate any money and that donating would not affect their chances of winning.

Participants randomized to the structured reflection condition first indicated how much (1 = *Not at all*; 7 = *Very much*) they believed their decision should be influenced by the “impact of donating to the [IRC’s] vaccination campaign on reducing COVID-19 risk among refugees” and “the economic benefit you would gain from receiving this prize money for personal economic relief.” Participants in the control condition rated their personal criteria after making their donation decision.

Participants reported that both reducing the COVID-19 risk among refugees (*M* = 3.77, *SD* = 2.06) and their personal economic relief (*M* = 3.86, *SD* = 2.06) should influence their donation decision. The two criteria were moderately correlated (*r* = 0.36, *P* < 0.001), were not significantly different from each other (*B* = −0.06, *SE* = 0.10, *CI* = [−0.26, 0.13], *P* = 0.533), and the difference did not vary by condition (*B* = −0.05, *SE* = 0.14, *CI* = [−0.33 , 0.22], *P* = 0.700, Supplementary Material, Table S12).

As predicted, participants in the structured reflection condition were more likely to donate (78%) than were participants in the control condition (72%, *B* = 0.28, *SE* = 0.14, *OR* = 1.32, *CI* = [1.00, 1.75], *P* = 0.047, Supplementary Material Table S13). We regressed the amount donated on condition (control = -½ and structured reflection = ½), ratings of each personal criteria (grand mean-centered), and interactions between condition and each rating. Participants in the structured reflection condition donated more (*M* = ${\$}$33.56, *SD* = ${\$}$33.49) compared with the control condition (*M* = ${\$}$28.73, *SD* = ${\$}$31.93; *B* = 3.71, *SE *= 1.62, *CI* = [0.53, 6.80], *P *= 0.022). These results indicate that structured reflection on personal criteria leads people to make donations that reduce others’ health risks.

Participants’ donation decisions were predicted both by their criteria about reducing the risk of COVID-19 among refugees (*B* = 9.77, *SE* = 0.60, *CI* = [8.59, 10.94], *P* < 0.001) and personal economic benefit (*B* = −2.99, *SE* = 0.60, *CI* = [−4.17, −1,81], *P* < 0.001). These correlations did not significantly differ across condition (refugee risk interaction: *B* = 0.26, *SE =* 0.84*, CI* = [−1.39, 1.92], *P *= 0.753; personal benefit interaction: *B* = 0.44, *SE =* 0.85*, CI* = [−1.22, 2.10], *P *= 0.606, Supplementary Material Table S14). As in experiment 2 (and unlike experiments 1 and 3), the absence of an interaction might be because the two criteria were positively correlated, and people have difficulty assigning different weights to correlated cues (54).

**Fig. 2.**
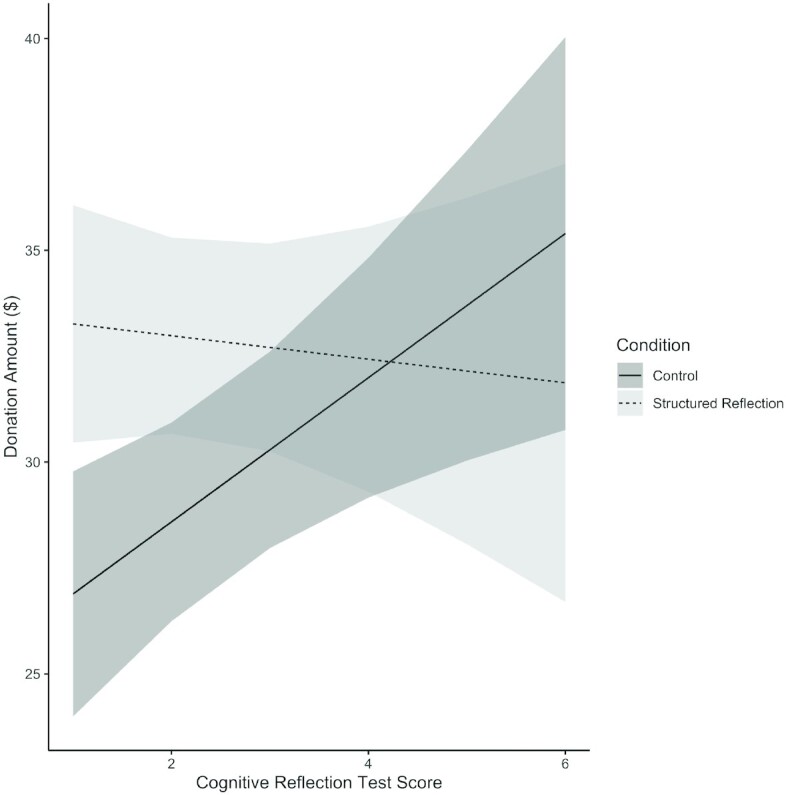
Estimated effect of cognitive reflection score on donations (of up to ${\$}$100) to the International Rescue Committee among participants randomized to the control condition (solid line with dark gray shade) and to the structured reflection condition (dashed line with light gray shade). Shaded regions represent 95% *CI*s around the estimates.

Finally, cognitive reflection, measured with six items (*M* = 2.43, *SD* = 1.88), moderated the effect of structured reflection (Fig. 2). When added to the regression analyses above, cognitive reflection scores predicted higher donations to provide COVID-19 vaccines for refugees (*B* = 1.70, *SE* = 0.60, *CI* = [0.52, 2.87], *P* = 0.005). There was also an interaction between cognitive reflection and condition (*B* = −2.08, *SE* = 0.88, *CI* = [−3.78, −0.32], *P* = 0.020). Structured reflection increased donations among participants with lower cognitive reflection scores (estimated at −1 SD, *B* = 7.35, *SE* = 2.32, *CI* = [2.80, 11.91], *P* = 0.002) more than among participants with higher cognitive reflection scores (estimated at + 1 SD, *B* = −0.34, *SE* = 2.34, *CI* = [−4.94, 4.27], *P* = 0.886). Structured reflection on personal criteria thus had more impact among those who were not otherwise predisposed to engage in cognitive reflection.

## Discussion

This research demonstrated that structured reflection on personal criteria increases intentions and decisions to minimize others’ health risks. We suggest that structured reflection increases attention to underweighted considerations of public health and leads people to construe decisions relative to their personal criteria. Consistent with this explanation, in experiments 1 and 3, structured reflection increased the correlation between behavioral intentions and reported personal criteria. Instructions to effortfully deliberate did not yield the same effect, suggesting that structured reflection differs from deliberation. And the effect of structured reflection on donations was smaller among people who scored higher on cognitive reflection and are more inclined to reflect of their own accord.

What other factors might moderate the effect of structured reflection on personal criteria? In exploratory analysis, we examined four potential moderators (see OSF for details: https://osf.io/ewr7g/). In experiments 1 to 3, liberal political ideology and trust in experts, including the World Health Organization and Centers for Disease Control and Prevention, predicted greater intentions to reduce others’ COVID-19 health risks. Neither measure consistently moderated the effect of structured reflection, except for experiment 1, where the effect of structured reflection was smaller among more liberal respondents. This moderation is attributable to a ceiling effect because relatively liberal participants report greater intentions to reduce restaurant capacity and thus have less room for structured reflection to increase such intentions. In experiment 4, trust in science (61) and scientific literacy (62) were both associated with higher donations to provide COVID-19 vaccines. Scientific literacy (but not trust in science) moderated the structured reflection effect (*B* = −1.33, *SE* = 0.67, *CI* = [−2.65, −0.01], *P* = 0.049). When both cognitive reflection and scientific literacy, which were strongly correlated (*r* = 0.41, *P* < 0.001), were included in the same regression analysis, the interaction effect of scientific literacy was substantially reduced and nonsignificant (*B =* −0.72, *SE* = 0.72, *CI* = [−2.19, 0.73], *P* = 0.326) whereas the interaction effect of cognitive reflection was modestly reduced and marginally significant (*B =* −0.83, *SE* = 0.48, *CI* = [−1.77, 0.12], *P* = 0.086). These exploratory analyses suggest that individual differences in cognitive reflection have a unique moderating effect on structured reflection.

How much are the effects of structured reflection due to experimental demand and socially desirable responses? Such motives undoubtedly contribute to the overall effect. Yet they are unlikely to fully explain it. If demand and desirability influence reporting of personal criteria, they do not readily explain why reporting criteria beforehand would influence intentions or decisions. If demand and desirability increase consistency, it is not clear why structured reflection did not influence personal criteria in experiments 1, 2, or 4, where people could have revised their personal criteria to appear consistent. We sought to reduce demand and desirability in experiment 4, reminding participants they might personally need personal economic relief during the COVID-19 pandemic. Finally, demand and desirability do not readily explain why individuals who score relatively low in cognitive reflection are more affected by structured reflection, a pattern consistent with our explanation.

These findings emerged across four scenarios and seven countries that varied in cultural values and pandemic policies, implicating the robustness and generalizability of the findings (40, 41). The effect of structured reflection was largest in Israel, a country that had relatively low COVID-19 rates and few restrictions at the time. An important question for future research is whether structured reflection would increase weight for others’ health risks in countries with even greater cultural differences and pandemic policies that are represented here. It may be that structured reflection would have less impact in contexts with severely restrictive lockdowns, such as Shanghai, where there is little room for voluntarily restricting social contact, or in contexts with little or no COVID-19 prevalence, such as New Zealand.

Another question for future research is whether structured reflection would have similar effects in scenarios with more complex attribute structures. We designed the scenarios to reflect tradeoffs between imposing health risks on other people versus more prominent considerations like economic payoffs and personal risks. Although structured reflection increased intentions and decisions to minimize others’ health risks in all experiments, the procedure increased the correlation between those outcomes and personal criteria only in the experiments (1 and 3) where the two criteria were largely orthogonal. When the criteria were positively correlated (experiments 2 and 4), structured reflection did not increase the correlation between personal criteria and outcomes. As noted, it may be that people have more difficulty differentiating positively correlated and complex attributes (54).

Can people learn to apply structured reflection in everyday life? We believe that structured reflection is akin to “boosts” that increase people's ability to make decisions according to their personal beliefs and values (63). Structured reflection entails three questions: Would the behavior impose health risks on other people? How much should imposing health risks on other people influence the decision to pursue the behavior? And how much should the potential benefits influence the decision to pursue the behavior? People should choose to reduce public health risks when they answer “yes” to the first question and assign relatively greater weight in the second versus third question. We are optimistic that public health communicators can help people learn to practice structured reflection to reduce public health risks.

## Methods and Materials

### Participants

For experiments 1 and 2, data were collected August 2 - 20, 2020, across residents in seven countries (total *N* = 12,995, *N_female_* = 6,153, *N_male_ *= 5,865, *N_other_* = 36): the United States (*N* = 3,318), Italy (*N* = 1,586), South Korea (*N* = 1,484), Sweden (*N* = 1,589), the United Kingdom (*N* = 1,520), Israel (*N* = 1,958), and Brazil (*N* = 1,500). We excluded four participants for implausibly high ages (>120). For experiments 1 and 2, resulting age ranged [18, 93], *M_age_* = 45.46, *SD_age_* = 15.61 and [18, 87], *M_age_* = 45.43, *SD_age_* = 15.55. Participants were randomly assigned to experiment 1 (*N* = 6,216) or experiment 2 (*N* = 6,070). In experiment 3, we recontacted US residents (*N *= 2,462) from the first two experiments (age range [18, 89], *M_age_ *= 49.95; *SD_age_ *= 15.04). As part of an unrelated study (64), participants were randomly assigned to one of three waves of data collection: October 30 to November 4, 2020 November 4 to 9, and November 9 to 15, 2020. We excluded 28 participants from experiment 1, 37 participants from experiment 2, and 4 participants from experiment 3, who did not respond to relevant measures. Finally, for experiment 4, we recontacted US residents (*N *= 1,080) from experiments 1 and 2 (we did not collect age data). We collected data from March 15 to April 7, 2020.

Samples were drawn from online panels in each country. Sampling quotas for age and gender were used to select respondents who were financially compensated for participation, except in the Swedish sample. Surveys were completed online and administered in each country's official or national language. Surveys were adapted from English by the authors who are fluent in both English and the target language and who have expertise in the cultural and political contexts of each country.

### Data analysis

We analyzed data in experiments 1 and 2 using mixed-effects regression models, allowing intercepts and slopes for all main effects to vary randomly across countries. Some models failed to converge when estimating random slope–intercept covariances, so they were reestimated, excluding those covariances. We calculated *P*values with Satterthwaite's approximation for degrees of freedom (65).

For the cross-country comparisons, we estimated a linear regression model with structured reflection, a set of six deviation-coded country contrasts, and the interaction between structured reflection and country codes as predictors. The coefficients correspond to interactions between the country codes and structured reflection conditions.

We analyzed data in experiment 3 with two orthogonal contrast codes for condition, one comparing the structured reflection condition against the average of the control and deliberation conditions (control = −1/3, deliberation = −1/3, structured reflection = 2/3), and one comparing the control and deliberation conditions (structured reflection = 0, control = −½, deliberation = ½) These contrasts were each interacted with ratings of personal criteria. We analyzed experiment 4 data using regression models with a condition contrast (control = −½ and structured reflection = ½).

### Scenarios, manipulations, and measures

In experiments 1 to 3, participants read scenarios that included considerations about minimizing other people's COVID-19 health risks by reducing social contact, as well as economic loss (experiment 1), personal health risks (experiment 2), and enjoyment of holiday socializing with family (experiment 3). In experiment 4, participants donated to provide COVID-19 vaccines to refugees, keeping the remainder for themselves.

***Experiment 1***: Suppose you are the owner of a small restaurant. Your restaurant has been running at full capacity throughout the last few weeks. However, there has recently been a spike of cases of the Coronavirus in your area, prompting your government to recommend that restaurants reduce their capacity to 50%. The goal of this recommendation is to minimize the spread of the virus; however, scaling down to 50% capacity would lead to a significant loss in your income, making it harder for you to afford your basic needs. You are trying to decide whether to keep your restaurant running at full capacity or reducing it to 50% capacity.
***Experiment 2***: One of your close friends has recently asked you, along with 50 other people, to come over to their house for a gathering to celebrate their birthday. Your local government has no laws against gatherings but recommends that people avoid meeting with more than 10 people at a time to reduce the spread of the Coronavirus. Still, attending this gathering would be a welcome opportunity to socialize and celebrate with your friend after months of limited social contact.
***Experiment 3***: Countries around the world have begun lifting legal restrictions on social and economic activities. Nonetheless, many experts recommend that people continue to take cautionary measures to prevent the spread of the novel Coronavirus. For a few months, one of your family members has been planning a Thanksgiving gathering and has invited you as well as 30 other family members (including several children and older adults) to this gathering. However, your family member is now considering canceling this gathering due to the pandemic and has asked you to decide whether it should be canceled. If the Thanksgiving gathering takes place, there is some probability that you or other family members may contract COVID-19 at the gathering. Still, attending this gathering would be a welcome opportunity to socialize and celebrate with your family after months of limited social contact. As a result, you are trying to decide whether to cancel the gathering.
***Experiment 4***: The COVID-19 pandemic has impacted people across the globe in many ways. In addition to the negative consequences for health and well-being, COVID-19 has negatively impacted most economies around the world, causing financial hardships for many. Considering these issues, we will randomly select one participant from this study to receive a ${\$}$100 economic relief prize as a bonus for participating. . . .The study participant who wins the ${\$}$100 prize will also have the opportunity to donate some portion of the ${\$}$100 prize money to the International Rescue Committe’s (IRC) vaccination campaign. The IRC is a non-profit that helps people affected by humanitarian crises to survive, recover, and rebuild their lives. The IRC's vaccination campaign provides COVID-19 vaccines to refugees around the world, who have limited access to vaccinations and medical care. You will indicate how much of the ${\$}$100 prize you want to keep for yourself as economic relief and how much you want to donate to the IRC's vaccination campaign. If you are chosen as a winner, we will allocate funds to you as personal economic relief funds and as a donation to the IRC based on what you tell us. Note that you are not required to donate any amount of money to the IRC's vaccination campaign, and your decision will not in any way affect your chances of winning the prize.

In experiments 1 to 3, after reading the scenario, participants randomized to the control conditions reported the relevant likelihood they would restrict restaurant capacity, attend the birthday party, or cancel Thanksgiving (1 = *Extremely unlikely;7 = Extremely likely*). In experiment 4, participants used a sliding scale to indicate how much of the ${\$}$100 cash prize they would donate to the IRC, keeping the rest for themselves. Within each experiment, before answering these questions, participants randomized to the structured reflection conditions read statements providing structure and two accompanying attributes relevant to the scenario before indicating how much (1 = *Not at all*; 7 = *Extremely* or *Very much*) each attribute should influence their decision:

***Experiment 1***: Keeping your business running as usual would allow you to maintain your current stream of income, thus helping you pay for your basic expenses. However, by keeping your business open at full capacity, your restaurant could potentially contribute to the increased spread of COVID-19.
How much should the economic losses associated with reducing restaurant capacity influence your decision to decrease working capacity by 50%?
How much should the risk that COVID-19 would spread among your employees and the patrons of your restaurant influence your decision to decrease working capacity by 50%?
***Experiment 2***: When deciding whether to attend this gathering, you might have to consider several different factors. Attending this gathering would increase your risk of contracting COVID-19. This means that you would have an increased likelihood of experiencing severe symptoms from COVID-19, as well as increased likelihood of spreading this disease to others that you interact with in your daily life.
How much should your likelihood of spreading COVID-19 influence your decision to attend the gathering?
How much should your likelihood of experiencing severe symptoms from COVID-19 influence your decision to attend the gathering?
***Experiment 3***: When deciding whether to cancel the Thanksgiving gathering, two main considerations are the likelihood that COVID-19 may spread among family members, and the family connection and satisfaction people would experience from attending this gathering.
How much should the likelihood that COVID-19 may spread among family members (including you and others attending) influence your decision of whether to cancel the gathering?
How much should the family connection and satisfaction that you and other family members would experience in this gathering influence your decision of whether to cancel the gathering?
***Experiment 4***: Think about the economic benefit you would gain from receiving this prize money as personal economic relief. That is, consider how much receiving some or all of the ${\$}$100 would help meet your needs, wants, or serve as a financial cushion. How much should the economic benefit you would gain from receiving this prize money for personal economic relief influence your decision to donate some or all of this money?
Think about the impact that money donated to the International Rescue Committe’s vaccination campaign would have on reducing COVID-19 risk among targeted refugees and their communities. That is, consider the impact that vaccine donations would have on reducing the spread and health consequences of COVID-19 among refugees and the communities they live in. How much should the impact of donating to the International Rescue Committe’s vaccination campaign on reducing COVID-19 risk among refugees and their communities influence your decision to donate some or all this money?

Participants in the control conditions read the above statements and reported their personal criteria after stating their intentions or decisions. In addition, before stating the likelihood of canceling Thanksgiving, participants in experiment 3 who were randomized to the deliberation condition were asked to “consider your decision carefully and try to generate clear reasons for why you should or should not cancel the Thanksgiving gathering” before reporting how likely they would be to cancel the gathering. The prompt explicitly asked participants to think carefully and rationally.

Experiment 4 included a six-item measure of cognitive reflection (66), comprising three numerical items and three nonnumerical items such as, “If you're running a race and you pass the person in second place, what place are you in?” (*α* = 0.75).

## Ethics Approval Statement

The Institutional Review Board at the University of Colorado categorized the study as exempt (Protocol 20-0197).

## Funding

This work was supported by the National Science Foundation grants SES: 2029183 to L.V.B. and 1757315 to P.S.

## Authors' Contributions

J.R., M.D.G., and L.V.B. wrote the initial manuscript. All authors conceptualized the study design, organized data collection, and reviewed versions of the manuscript. A.F. supervised data collection and organized data. J.R., M.D.G., E.J.P., and L.V.B. conducted and reviewed data analysis.

## Supplementary Material

pgac218_Supplemental_File

## Data Availability

Materials, data, analysis scripts, and results are available on Open Science Framework at https://osf.io/ewr7g/.
