# Correction to: Structured reflection increases intentions to reduce other people’s health risks during COVID-19

**DOI:** 10.1093/pnasnexus/pgad472

**Published:** 2024-01-30

This is a correction to: Jairo Ramos, Marrissa D Grant, Stephan Dickert, Kimin Eom, Alex Flores, Gabriela M Jiga-Boy, Tehila Kogut, Marcus Mayorga, Eric J Pedersen, Beatriz Pereira, Enrico Rubaltelli, David K Sherman, Paul Slovic, Daniel Västfjäll, Leaf Van Boven, Structured reflection increases intentions to reduce other people’s health risks during COVID-19, *PNAS Nexus*, Volume 1, Issue 5, November 2022, pgac218, https://doi.org/10.1093/pnasnexus/pgac218

During a retroactive audit conducted by *PNAS Nexus*, it was discovered that this paper was missing a statement acknowledging compliance with the *PNAS Nexus* Human and Animal Participants and Clinical Trials policy:

The Institutional Review Board at the University of Colorado categorized the study as exempt (Protocol 20-0197).

This error has been corrected in the original article.

