# Supplementary material for: Structured reflection increases intentions to reduce other people’s health risks during COVID-19
Source: PNAS Nexus. 2022 Oct 3;1(5):pgac218. doi: 10.1093/pnasnexus/pgac218 (PMC9802473; doi:10.1093/pnasnexus/pgac218)
Supplement: pgac218_Supplemental_File [file pgac218_supplemental_file.docx]

**
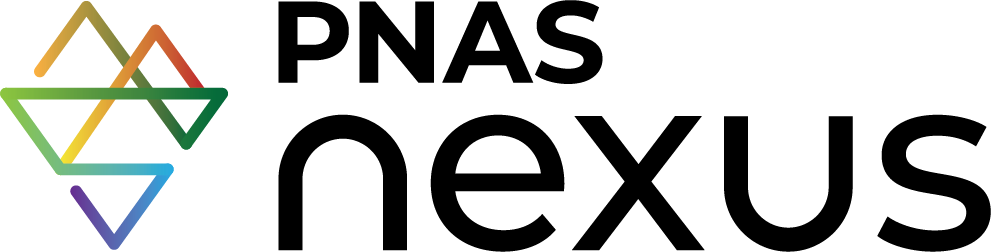
**

**Structured Reflection Increases Intentions to Reduce Other People’s Health Risks During Covid-19**

Jairo Ramos^1^, Marrissa D. Grant^1^, Stephan Dickert^2^, Kimin Eom^3^, Alex Flores^1^, Gabriela M. Jiga-Boy^4^, Tehila Kogut^5^, Marcus Mayorga^6^, Eric J. Pedersen^1^, Beatriz Pereira^7^, Enrico Rubaltelli^8^, David K. Sherman^9^, Paul Slovic^6^, Daniel Västfjäll^10^, Leaf Van Boven^1,×^

^1^ Department of Psychology and Neuroscience, University of Colorado Boulder

^2^ School of Business and Management, Queen Mary University of London, London, England; Department of Psychology, University of Klagenfurt, Klagenfurt, Austria

^3^ School of Social Sciences, Singapore Management University, Singapore

^4^ School of Psychology, Swansea University, Swansea, United Kingdom

^5^ Department of Education, Ben-Gurion University of the Negev, Be’er Sheva, Israel

^6^ Decision Research; University of Oregon

^7^ Department of Marketing, Ivy College of Business, Iowa State University

^8^ Department of Developmental and Social Psychology, University of Padua, Padova, Italy

^9^ Department of Psychological and Brain Sciences, University of California, Santa Barbara,

^10^ Department of Behavioural Sciences and Learning, Division of Psychology, Linköping University, Linköping, Sweden

Corresponding author: Leaf Van Boven

**Email:** [vanboven@colorado.edu](mailto:vanboven@colorado.edu)

**This PDF file includes:** Tables S1 to S14

**Table S1.** Pandemic status and Hofstede cultural dimension for each of seven countries form which participants were sampled in Experiments 1 and 2.

|  | **Pandemic Status (August 1, 2021)** | | | **Hofstede Cultural Dimensions** |
| --- | --- | --- | --- | --- |
| **Country** | Oxford’s Stringency Index | Covid-19 Cases (per million people) | Death Rate (per million people) | Individualism vs. Collectivism |
| Brazil | 72.69 | 210.61 | 7.36 | 38 |
| Israel | 52.78 | 180.61 | 0.02 | 54 |
| Italy | 52.78 | 4.65 | 0.51 | 76 |
| South Korea | 53.24 | 0.60 | 0.04 | 18 |
| Sweden | 59.26 | 22.07 | 0.18 | 71 |
| United Kingdom | 59.26 | 22.07 | 0.18 | 71 |
| United States | 69.91 | 11.14 | 0.24 | 89 |

*Note.* Oxford’s Covid-19 stringency index is a measure of the strictness of ‘lockdown style’ restrictions on behavior computed by the [Oxford Covid-19 Government Response Tracker](https://www.bsg.ox.ac.uk/research/research-projects/covid-19-government-response-tracker) (1-100). Pandemic Status statistics were retrieved from [Ourworldindata.com](http://ourworldindata.com/) and correspond to figures reported as of August 1, 2020. Hofstede’s Individualism vs. Collectivism scale (1-100) is measured as part of Hofstede’s six-dimension model of national culture. Higher numbers indicate higher individualism and lower collectivism. Details on the model and empirical validation procedures are available in this report: [Dimensionalizing Culture: the Hofstede model in context](http://scholarworks.gvsu.edu/cgi/viewcontent.cgi?article=1014&context=orpc).

**Table S2.** Experiments 1 and 2: Means and standard deviations for the two ratings of personal criteria and behavioral intentions for the structured reflection and control conditions.

|  | **Experiment 1: Restaurant Business Capacity** | | | | | |
| --- | --- | --- | --- | --- | --- | --- |
|  | Risk of  Spreading COVID-19 | | Risk of  Economic Losses | | Intention to Decrease Restaurant Capacity | |
| Country | Structured Reflection | Control | Structured Reflection | Control | Structured Reflection | Control |
|  | M(SD) | M(SD) | M(SD) | M(SD) | M(SD) | M(SD) |
| Brazil | 5.10(1.68) | 5.25(1.73) | 4.14(1.84) | 4.24(1.72) | 5.56(1.75) | 5.32(1.69) |
| Israel | 5.30(1.36) | 5.09(1.49) | 4.86(1.65) | 4.63(1.60) | 4.88(1.60) | 4.15(1.73) |
| Italy | 5.03(1.38) | 4.82(1.54) | 4.33(1.59) | 4.14(1.52) | 4.97(1.64) | 4.67(1.73) |
| South Korea | 4.61(1.25) | 4.51(1.48) | 4.29(1.28) | 4.30(1.30) | 4.51(1.36) | 4.48(1.49) |
| Sweden | 5.06(1.27) | 4.98(1.31) | 3.76(1.58) | 3.83(1.44) | 5.47(1.52) | 5.27(1.67) |
| United Kingdom | 5.61(1.42) | 5.61(1.52) | 3.81(1.61) | 3.87(1.65) | 5.58(1.45) | 5.27(1.53) |
| United States | 5.14(1.68) | 5.08(1.72) | 4.18(1.75) | 4.09(1.69) | 5.28(1.73) | 5.06(1.78) |
|  | **Experiment 2: Social Gathering** | | | | | |
|  | Risk of  Spreading COVID-19 | | Risk of Experiencing  Severe COVID-19 Symptoms | | Intention to Attend Gathering | |
|  | Structured Reflection | Control | Structured Reflection | Control | Structured Reflection | Structured Reflection |
| Country | M(SD) | M(SD) | M(SD) | M(SD) | M(SD) | M(SD) |
| Brazil | 4.91(2.07) | 5.05(2.07) | 4.94(2.09) | 5.02(2.05) | 2.72(1.86) | 2.80(1.91) |
| Israel | 5.55(1.56) | 5.69(1.49) | 5.44(1.64) | 5.50(1.63) | 3.26(1.85) | 3.66(1.93) |
| Italy | 5.33(1.69) | 5.37(1.71) | 5.37(1.58) | 5.38(1.69) | 2.72(1.84) | 2.98(1.95) |
| South Korea | 4.57(1.86) | 4.52(1.62) | 4.66(1.83) | 4.57(1.59) | 2.91(1.60) | 3.00(1.57) |
| Sweden | 5.50(1.36) | 5.23(1.55) | 5.19(1.51) | 4.79(1.67) | 2.14(1.75) | 2.37(1.89) |
| United Kingdom | 6.13(1.35) | 5.90(1.47) | 5.82(1.57) | 5.60(1.62) | 1.98(1.53) | 2.30(1.79) |
| United States | 5.53(1.85) | 5.32(1.97) | 5.52(1.86) | 5.32(1.99) | 2.35(1.83) | 2.67(2.07) |

**Table S3.** Experiment 1: Coefficients for mixed effects logistic regression estimating Intentions to reduce business capacity as a binned dichotomous outcome.

|  | Intentions to reduce business capacity (Discrete) | | | |
| --- | --- | --- | --- | --- |
|  | *OR* | *SE* | *CI* | *p* |
| Intercept | 2.22 | 0.39 | 1.57 – 3.13 | **<0.001** |
| Condition | 1.39 | 0.14 | 1.14 – 1.69 | **0.001** |

**Table S4.** Experiment 1: Coefficients for mixed effects regressions estimating intentions to reduce business capacity.

|  | Intentions to reduce capacity | | | | Intentions to reduce capacity | | | |
| --- | --- | --- | --- | --- | --- | --- | --- | --- |
|  | *B* | *SE* | *CI* | *p* | *B* | *SE* | *CI* | *p* |
| Intercept | 5.04 | 0.12 | 4.81 – 5.26 | **<0.001** | 5.03 | 0.16 | 4.72 – 5.34 | **<0.001** |
| Condition | 0.26 | 0.07 | 0.11 – 0.40 | **0.014** | 0.29 | 0.08 | 0.13 – 0.45 | **0.012** |
| Econ. | -0.14 | 0.01 | -0.16 – -0.12 | **<0.001** |  |  |  |  |
| Spread | 0.51 | 0.01 | 0.48 – 0.53 | **<0.001** |  |  |  |  |
| Cond x Econ. | -0.05 | 0.02 | -0.10 – -0.01 | **0.018** |  |  |  |  |
| Cond x Spread | 0.08 | 0.02 | 0.03 – 0.13 | **0.001** |  |  |  |  |

**Table S5.** Experiment 1: Coefficients for linear regressions estimating Intentions to reduce business capacity treating country as a fixed factor.

|  | Intentions to reduce business capacity | | | |
| --- | --- | --- | --- | --- |
| Predictors | *B* | *SE* | *CI* | ***p*** |
| Intercept | 5.01 | 0.02 | 4.97 – 5.05 | **<0.001** |
| Condition | 0.27 | 0.04 | 0.19 – 0.35 | **<0.001** |
| Econ. | -0.12 | 0.01 | -0.15 – -0.09 | **<0.001** |
| Spread | 0.45 | 0.01 | 0.42 – 0.47 | **<0.001** |
| UK | 0.10 | 0.05 | -0.00 – 0.20 | 0.057 |
| Korea | -0.43 | 0.05 | -0.53 – -0.32 | **<0.001** |
| Italy | -0.09 | 0.05 | -0.19 – 0.01 | 0.07 |
| Sweden | 0.28 | 0.05 | 0.19 – 0.38 | **<0.001** |
| Israel | -0.41 | 0.05 | -0.50 – -0.31 | **<0.001** |
| Brazil | 0.40 | 0.05 | 0.30 – 0.49 | **<0.001** |
| Condition × Econ. | -0.07 | 0.03 | -0.12 – -0.02 | **0.006** |
| Condition × Spread | 0.06 | 0.03 | 0.00 – 0.11 | **0.037** |
| Condition × UK | 0.03 | 0.10 | -0.17 – 0.23 | 0.771 |
| Condition × Korea | -0.20 | 0.11 | -0.41 – 0.01 | 0.057 |
| Condition × Italy | -0.03 | 0.10 | -0.22 – 0.17 | 0.788 |
| Condition × Sweden | -0.17 | 0.10 | -0.36 – 0.02 | 0.076 |
| Condition × Israel | 0.42 | 0.10 | 0.22 – 0.61 | **<0.001** |
| Condition × Brazil | 0.02 | 0.10 | -0.16 – 0.21 | 0.801 |
| Econ. × UK | -0.14 | 0.03 | -0.20 – -0.08 | **<0.001** |
| Econ. × Korea | 0.27 | 0.04 | 0.19 – 0.35 | **<0.001** |
| Econ. × Italy | -0.05 | 0.03 | -0.12 – 0.01 | 0.107 |
| Econ. × Sweden | -0.13 | 0.03 | -0.19 – -0.07 | **<0.001** |
| Econ. × Israel | -0.11 | 0.03 | -0.17 – -0.06 | **<0.001** |
| Econ. × Brazil | 0.19 | 0.03 | 0.13 – 0.25 | **<0.001** |
| Spread × UK | -0.01 | 0.03 | -0.07 – 0.06 | 0.814 |
| Spread × Korea | -0.25 | 0.04 | -0.33 – -0.17 | **<0.001** |
| Spread × Italy | 0.13 | 0.03 | 0.06 – 0.20 | **<0.001** |
| Spread × Sweden | -0.10 | 0.04 | -0.17 – -0.03 | **0.007** |
| Spread × Israel | 0.08 | 0.03 | 0.01 – 0.14 | **0.019** |
| Spread × Brazil | 0.04 | 0.03 | -0.02 – 0.10 | 0.231 |
| (Condition × Econ.) × UK | 0.02 | 0.06 | -0.10 – 0.14 | 0.693 |
| (Condition × Econ.) × Korea | -0.04 | 0.08 | -0.20 – 0.12 | 0.634 |
| (Condition × Econ.) × Italy | 0.05 | 0.06 | -0.08 – 0.17 | 0.471 |
| (Condition × Econ.) × Sweden | -0.06 | 0.06 | -0.18 – 0.06 | 0.349 |
| (Condition × Econ.) × Israel | 0.02 | 0.06 | -0.10 – 0.13 | 0.788 |
| (Condition × Econ.) × Brazil | 0.04 | 0.06 | -0.07 – 0.16 | 0.459 |
| (Condition × Spread) × UK | -0.11 | 0.07 | -0.24 – 0.03 | 0.116 |
| (Condition × Spread) × Korea | 0.02 | 0.08 | -0.13 – 0.18 | 0.792 |
| (Condition × Spread) × Italy | 0.10 | 0.07 | -0.03 – 0.24 | 0.143 |
| (Condition × Spread) × Sweden | -0.20 | 0.07 | -0.35 – -0.06 | **0.005** |
| (Condition × Spread) × Israel | -0.02 | 0.07 | -0.15 – 0.11 | 0.769 |
| (Condition × Spread) × Brazil | 0.20 | 0.06 | 0.08 – 0.32 | **0.001** |

**Table S6.** Experiment 2: Coefficients for mixed effects logistic regression estimating intentions not to attend a social gathering binned as a dichotomous outcome.

|  | Intentions to not attend a social gathering (Discrete) | | | |
| --- | --- | --- | --- | --- |
|  | *OR* | *SE* | *CI* | *p* |
| Intercept | 2.30 | 0.38 | 1.66 – 3.19 | **<0.001** |
| Condition | 1.24 | 0.07 | 1.11 – 1.39 | **<0.001** |

**Table S7.** Experiment 2: Coefficients for mixed effects regressions estimating Intentions to attend a social gathering.

|  | Intentions to attend a social gathering | | | | Intentions to attend a social gathering | | | |
| --- | --- | --- | --- | --- | --- | --- | --- | --- |
|  | *B* | *SE* | *CI* | *p* | *B* | *SE* | *CI* | *p* |
| (Intercept) | 2.69 | 0.17 | 2.36 – 3.01 | **<0.001** | 2.7 | 0.17 | 2.37 – 3.04 | **<0.001** |
| Condition | -0.2 | 0.04 | -0.29 – -0.12 | **0.008** | -0.26 | 0.05 | -0.35 – -0.16 | **<0.001** |
| Spread | -0.32 | 0.02 | -0.36 – -0.28 | **<0.001** |  |  |  |  |
| Personal | -0.17 | 0.02 | -0.20 – -0.13 | **<0.001** |  |  |  |  |
| Condition × Spread | -0.06 | 0.04 | -0.13 – 0.01 | 0.082 |  |  |  |  |
| Condition × Personal | 0.02 | 0.04 | -0.05 – 0.09 | 0.521 |  |  |  |  |

**Table S8.** Experiment 2: Coefficients for regressions estimating Intentions to attend a social gathering treating country as a fixed factor.

|  | Intentions to attend a social gathering | | | |
| --- | --- | --- | --- | --- |
| Predictors | B | SE | CI | p |
| Intercept | 2.76 | 0.02 | 2.72 – 2.81 | **<0.001** |
| Condition | -0.15 | 0.05 | -0.24 – -0.06 | **0.001** |
| Spread | -0.33 | 0.02 | -0.37 – -0.29 | **<0.001** |
| Personal | -0.16 | 0.02 | -0.20 – -0.12 | **<0.001** |
| UK | -0.25 | 0.06 | -0.37 – -0.13 | **<0.001** |
| Korea | 0.17 | 0.06 | 0.04 – 0.29 | **0.009** |
| Italy | 0.10 | 0.06 | -0.01 – 0.21 | 0.078 |
| Sweden | -0.55 | 0.06 | -0.66 – -0.44 | **<0.001** |
| Israel | 0.83 | 0.05 | 0.72 – 0.93 | **<0.001** |
| Brazil | -0.09 | 0.06 | -0.20 – 0.02 | 0.108 |
| Condition × Spread | -0.09 | 0.04 | -0.17 – -0.01 | **0.022** |
| Condition × Personal | -0.01 | 0.04 | -0.08 – 0.07 | 0.85 |
| Condition × UK | 0.07 | 0.12 | -0.16 – 0.31 | 0.549 |
| Condition × Korea | 0.10 | 0.13 | -0.15 – 0.35 | 0.42 |
| Condition × Italy | -0.13 | 0.11 | -0.35 – 0.09 | 0.233 |
| Condition × Sweden | 0.15 | 0.11 | -0.06 – 0.37 | 0.17 |
| Condition × Israel | -0.23 | 0.11 | -0.44 – -0.03 | **0.026** |
| Condition × Brazil | 0.06 | 0.11 | -0.16 – 0.28 | 0.603 |
| Spread × UK | -0.15 | 0.05 | -0.25 – -0.04 | **0.005** |
| Spread × Korea | 0.34 | 0.06 | 0.22 – 0.46 | **<0.001** |
| Spread × Italy | -0.12 | 0.05 | -0.22 – -0.03 | **0.012** |
| Spread × Sweden | -0.14 | 0.05 | -0.23 – -0.04 | **0.004** |
| Spread × Israel | -0.06 | 0.04 | -0.14 – 0.03 | 0.176 |
| Spread × Brazil | 0.16 | 0.04 | 0.08 – 0.25 | **<0.001** |
| Personal × UK | 0.03 | 0.05 | -0.06 – 0.12 | 0.539 |
| Personal × Korea | 0.10 | 0.06 | -0.02 – 0.23 | 0.104 |
| Personal × Italy | -0.02 | 0.05 | -0.12 – 0.08 | 0.744 |
| Personal × Sweden | -0.07 | 0.04 | -0.15 – 0.02 | 0.135 |
| Personal × Israel | -0.03 | 0.04 | -0.11 – 0.04 | 0.387 |
| Personal × Brazil | 0.08 | 0.04 | -0.00 – 0.16 | 0.052 |
| (Condition × Spread) × UK | 0.00 | 0.11 | -0.21 – 0.20 | 0.967 |
| (Condition × Spread) × Korea | 0.25 | 0.13 | -0.00 – 0.49 | 0.052 |
| (Condition × Spread) × Italy | -0.04 | 0.10 | -0.24 – 0.15 | 0.656 |
| (Condition × Spread) × Sweden | -0.22 | 0.10 | -0.41 – -0.03 | **0.02** |
| (Condition × Spread) × Israel | -0.19 | 0.08 | -0.35 – -0.02 | **0.028** |
| (Condition × Spread) × Brazil | 0.07 | 0.08 | -0.09 – 0.23 | 0.399 |
| (Condition × Personal) × UK | -0.05 | 0.09 | -0.23 – 0.14 | 0.619 |
| (Condition × Personal) × Korea | -0.12 | 0.13 | -0.37 – 0.13 | 0.357 |
| (Condition × Personal) × Italy | 0.05 | 0.10 | -0.15 – 0.25 | 0.625 |
| (Condition × Personal) × Sweden | 0.01 | 0.09 | -0.16 – 0.18 | 0.926 |
| (Condition × Personal) × Israel | 0.05 | 0.08 | -0.11 – 0.20 | 0.552 |
| (Condition × Personal) × Brazil | 0.10 | 0.08 | -0.06 – 0.26 | 0.222 |

**Table S9.** Experiment 3: Means and standard deviations for the two ratings of personal criteria and behavioral intentions for the structured reflection (SR), control, and deliberation conditions

| **Experiment 3: Thanksgiving Gathering Scenario** | | | | | | | | |
| --- | --- | --- | --- | --- | --- | --- | --- | --- |
| Risk of Spreading  Covid-19 | | | Satisfaction Spending  Time with Family | | | Likelihood of  Canceling Gathering | | |
| SR | Control | Deliberation | SR | Control | Deliberation | SR | Control | Deliberation |
| M(SD) | M(SD) | M(SD) | M(SD) | M(SD) | M(SD) | M(SD) | M(SD) | M(SD) |
| 5.56 (1.82) | 5.35 (1.92) | 5.41 (1.95) | 3.95  (1.89) | 4.05  (1.94) | 4.10 (1.87) | 5.22  (2.04) | 4.88 ( (2.19) | 4.93 (2.16) |

**Table S10.** Experiment 3: Coefficients for logistic regression estimating Intentions to cancel a Thanksgiving gathering binned as a dichotomous outcome.

|  | Intentions to cancel a Thanksgiving gathering (Discrete) | | | |
| --- | --- | --- | --- | --- |
|  | *OR* | *SE* | *CI* | *p* |
| Intercept | 1.6 | 0.07 | 1.47 – 1.74 | **<0.001** |
| Other vs. SR | 1.23 | 0.11 | 1.03 – 1.47 | **0.020** |
| Control vs. Deliberation | 0.99 | 0.1 | 0.81 – 1.20 | 0.899 |

**Table S11.** Experiment 3: Coefficients for linear regressions estimating Intentions to cancel a Thanksgiving gathering.

|  | Intentions to cancel a  Thanksgiving gathering | | | | Intentions to cancel a  Thanksgiving gathering | | | |
| --- | --- | --- | --- | --- | --- | --- | --- | --- |
|  | *B* | *SE* | *CI* | *p* | *B* | *SE* | *CI* | *P* |
| (Intercept) | 5.01 | 0.03 | 4.95 – 5.06 | **<0.001** | 5.01 | 0.04 | 4.92 – 5.09 | **<0.001** |
| Other vs. SR | 0.15 | 0.06 | 0.03 – 0.26 | **0.015** | 0.32 | 0.09 | 0.14 – 0.50 | **<0.001** |
| Control vs. Deliberation | 0.00 | 0.07 | -0.13 – 0.14 | 0.965 | 0.05 | 0.11 | -0.16 – 0.25 | 0.667 |
| Spread | 0.84 | 0.02 | 0.81 – 0.87 | **<0.001** |  |  |  |  |
| Satisfaction | -0.1 | 0.01 | -0.13 – -0.07 | **<0.001** |  |  |  |  |
| Other vs. SR × Spread | 0.11 | 0.03 | 0.05 – 0.18 | **<0.001** |  |  |  |  |
| Other vs. SR × Satisfaction. | -0.02 | 0.03 | -0.08 – 0.05 | 0.61 |  |  |  |  |
| Control vs. Deliberation × Spread | -0.05 | 0.04 | -0.12 – 0.02 | 0.142 |  |  |  |  |
| Control vs. Deliberation × Satisfaction. | -0.03 | 0.04 | -0.10 – 0.04 | 0.442 |  |  |  |  |
|  |  |  |  |  |  |  |  |  |

**Table S12.** Experiment 4: Means and standard deviations for the two ratings of personal criteria and donations (out of $100) for the structured reflection and control conditions

| **Experiment 4: Donations** | | | | | |
| --- | --- | --- | --- | --- | --- |
| Risk of Spreading  Covid-19 | | Personal Economic Relief | | Donation Amount | |
| SR | Control | SR | Control | SR | Control |
| M(SD) | M(SD) | M(SD) | M(SD) | M(SD) | M(SD) |
| 3.86 (2.09) | 3.68 (2.03) | 3.98 (2.08) | 3.75 (2.03) | 33.66 (33.49) | 28.73 (31.93) |

**Table S13.** Experiment 4: Coefficients for logistic regression estimating donations to the International Rescue Committee’s vaccination campaign binned as a dichotomous (zero vs. Non-zero amount) outcome.

|  | Donate to International Rescue Committee (Yes/No) | | | |
| --- | --- | --- | --- | --- |
|  | *OR* | *SE* | *CI* | *p* |
| Intercept | 3.03 | 0.21 | 2.64 – 3.48 | **<0.001** |
| Condition | 1.15 | 0.08 | 1.00 – 1.32 | **0.047** |

**Table S14.** Experiment 4: Coefficients for regressions estimating donations to the International Rescue Committee vaccination campaign.

|  | Donation Amount to IRC | | | | Donation Amount to IRC | | | |
| --- | --- | --- | --- | --- | --- | --- | --- | --- |
|  | *B* | *SE* | *CI* | *p* | *B* | *SE* | *CI* | *p* |
| Intercept | 31.19 | 0.81 | 29.60 – 32.78 | **<0.001** | 31.19 | 1.00 | 29.24 – 33.15 | **<0.001** |
| Condition | 1.86 | 0.81 | 0.26 – 3.45 | **0.022** | 2.46 | 1.00 | 0.51 – 4.42 | **0.014** |
| Spread | 9.9 | 0.42 | 9.08 – 10.73 | **<0.001** |  |  |  |  |
| Econ. | -2.77 | 0.42 | -3.60 – -1.94 | **<0.001** |  |  |  |  |
| Condition × Spread | 0.13 | 0.42 | -0.69 – 0.96 | 0.754 |  |  |  |  |
| Condition × Econ. | 0.22 | 0.42 | -0.61 – 1.05 | 0.606 |  |  |  |  |
